# Supplementary material for: Epidemiology of ovale, vivax and falciparum malaria in the highlands of Cameroon: An integrated community survey of human infection and vector abundance
Source: PLoS Negl Trop Dis. 2025 Oct 10;19(10):e0013549. doi: 10.1371/journal.pntd.0013549 (PMC12513583; doi:10.1371/journal.pntd.0013549)
Supplement: S1 Text — Table A. Sampling Weights. Table B. Malaria qPCR Detection Methods. Table C. P. falciparum Gametocyte Detection by qPCR Methods. Table D. P. ovale spp Gametocyte Detection by qPCR Methods. Table E. Limit of detection for P. ovale spp. gametocyte assay. Table F. Interassay and intra-assay coefficient of variation (CV) for male P. ovale gametocyte detection using pomget. Three serially diluted standards in triplicate across 5 different qPCR runs. Table G. Weighted bivariate and adjusted odds ratios with 95% CI. Table H. Anopheles abundance in Dschang neighborhoods. Fig A. Distribution of parasitemias for P. ovale spp, P. falciparum and P. vivax. Fig B. Correlation between gametocytemia and parasitemia in P. falciparum. Fig C. Limit of Detection (LOD) for pomget assay. A Probit analysis was conducted of replicate runs carried out using synthesized plasmids containing the pomget target sequence. An estimated LoD was calculated to be 281 transcript copies/uL (95% CI 162, 1310) based on the observed hit rate. Fig D. Mosquito capture time for indoor and outdoor collections across all households. (DOCX) [file pntd.0013549.s001.docx]

**Epidemiology of Relapsing and Falciparum Malaria in the Highlands of Cameroon: An Integrated Community Survey of Human Infection and Vector Abundance**

Samuel J. White, Valery P. K. Tchuenkam, Miriama Mbouh, Claudia Gaither, Aline Gaelle Bouopda-Tuedom, Belinda Claire Kiam, Zachary R. Popkin-Hall, Jacob M. Sadler, Kelly Carey-Ewend, Emily Hand, Miriam N. Ngum, Yannick N. Ngomsi, Jeffrey A Bailey, Darlin B. Kaunda, Lethicia Kamga Mafo, Giresse N. Lemogo, Clifford Dinka, Clifford A. Nsani, Michel Noubom, Varun Goel, Ibrahima Ibrahima, Janvier C. Onguene, Feng-Chang Lin, Jessica T. Lin, Rhoel R. Dinglasan, Sandrine E. Nsango, Jonathan J. Juliano, Innocent Mbulli Ali

Table A ……………………………………………………………..…………….. 2

Table B …………………………………………………………………..……….. 3

Table C ………………………………………………………………..………….. 4

Table D ………………………………………………………………..………….. 5

Table E ………………………………………………………………..………….. 6

Table F ………………………………………………………………..………….. 7

Table G ………………………………………………………………..………….. 8

Table H ……………………………………………………………..…………….. 9

Fig A .……………………………………………………………..…………… 10

Fig B .…………………………………………………………..……………… 11

Fig C .…………………………………………………………..……………… 12

Fig D .…………………………………………………………..……………… 13

**Table A. Sampling Weights**

| **Health Area** | **Village** | **Number of Clusters** | **Probability of Cluster Selected** | **Estimated Number of Households** | **Number of Households Selected** | **Probability of Household Selected** | **Weight** |
| --- | --- | --- | --- | --- | --- | --- | --- |
| Baleveng | Menka | 21 | 0.286 | 291 | 15 | 0.05 | 67.85 |
| Baleveng | Lepang | 21 | 0.286 | 282 | 10 | 0.04 | 98.84 |
| Baleveng | Dijo | 21 | 0.286 | 491 | 20 | 0.04 | 85.89 |
| Baleveng | Zem | 21 | 0.286 | 204 | 24 | 0.12 | 29.75 |
| Baleveng | Nza'ah | 21 | 0.286 | 565 | 28 | 0.05 | 70.68 |
| Baleveng | Melio | 21 | 0.286 | 180 | 1 | 0.01 | 628.60 |
| Doumbouo | Suella Batsingla | 20 | 0.2 | 280 | 13 | 0.05 | 107.69 |
| Doumbouo | Kamnick | 20 | 0.2 | 126 | 12 | 0.10 | 52.42 |
| Doumbouo | Tsing Saah | 20 | 0.2 | 334 | 12 | 0.04 | 139.08 |
| Doumbouo | Saah | 20 | 0.2 | 150 | 14 | 0.09 | 53.57 |
| Fialah Foréké | Zembing | 21 | 0.667 | 137 | 30 | 0.22 | 6.85 |
| Fialah Foréké | Mingmeto | 21 | 0.667 | 384 | 38 | 0.10 | 15.16 |
| Fialah Foréké | Femlah | 21 | 0.667 | 266 | 13 | 0.05 | 30.69 |
| Fialah Foréké | Mingou route | 21 | 0.667 | 454 | 10 | 0.02 | 68.10 |
| Fialah Foréké | Ngui 3 | 21 | 0.667 | 205 | 13 | 0.06 | 23.65 |
| Fialah Foréké | Vallee | 21 | 0.667 | 368 | 29 | 0.08 | 19.03 |
| Fialah Foréké | Lefock | 21 | 0.667 | 258 | 27 | 0.10 | 14.33 |
| Fialah Foréké | Ngui 1 | 21 | 0.667 | 488 | 25 | 0.05 | 29.28 |
| Fialah Foréké | Doungha | 21 | 0.667 | 548 | 18 | 0.03 | 45.67 |
| Fialah Foréké | Meka'a | 21 | 0.667 | 819 | 30 | 0.04 | 40.95 |
| Fialah Foréké | Toumeto | 21 | 0.667 | 305 | 12 | 0.04 | 38.13 |
| Fialah Foréké | Mechieu | 21 | 0.667 | 437 | 10 | 0.02 | 65.55 |
| Fialah Foréké | Ngui 2 | 21 | 0.667 | 344 | 12 | 0.03 | 43.00 |
| Fialah Foréké | King place | 21 | 0.667 | 270 | 16 | 0.06 | 25.31 |
| Fometa | Fiakop 2 | 16 | 0.375 | 205 | 22 | 0.11 | 24.85 |
| Fometa | Keleng 1 | 16 | 0.375 | 251 | 14 | 0.06 | 47.81 |
| Fometa | Touleppe | 16 | 0.375 | 195 | 14 | 0.07 | 37.14 |
| Fometa | Lefatsa | 16 | 0.375 | 204 | 15 | 0.07 | 36.27 |
| Fometa | Fialah | 16 | 0.375 | 289 | 17 | 0.06 | 45.33 |
| Fometa | Minpeuh | 16 | 0.375 | 159 | 16 | 0.10 | 26.50 |
| Mboua | Mboua | 15 | 0.267 | 303 | 16 | 0.05 | 71.02 |
| Mboua | Apang | 15 | 0.267 | 58 | 10 | 0.17 | 21.75 |
| Mboua | Dzifoda | 15 | 0.267 | 128 | 14 | 0.11 | 34.29 |
| Mboua | Fossong | 15 | 0.267 | 352 | 13 | 0.04 | 101.54 |
| Penka-Michel | Bagham | 19 | 0.421 | 116 | 13 | 0.11 | 21.19 |
| Penka-Michel | Iac | 19 | 0.421 | 121 | 15 | 0.12 | 19.16 |
| Penka-Michel | Ionako | 19 | 0.421 | 319 | 15 | 0.05 | 50.51 |
| Penka-Michel | Nylon | 19 | 0.421 | 178 | 18 | 0.10 | 23.49 |
| Penka-Michel | Plateau | 19 | 0.421 | 247 | 15 | 0.06 | 39.11 |
| Penka-Michel | Tami | 19 | 0.421 | 96 | 17 | 0.18 | 13.41 |
| Penka-Michel | Tergal | 19 | 0.421 | 130 | 14 | 0.11 | 22.05 |
| Penka-Michel | Hopital | 19 | 0.421 | 89 | 15 | 0.17 | 14.09 |
| Siteu | Tapale | 15 | 0.667 | 171 | 16 | 0.09 | 16.03 |
| Siteu | Nylon | 15 | 0.667 | 151 | 15 | 0.10 | 15.10 |
| Siteu | Tsinkop | 15 | 0.667 | 242 | 23 | 0.10 | 15.78 |
| Siteu | Tchouale 2 | 15 | 0.667 | 242 | 17 | 0.07 | 21.35 |
| Siteu | Madagascar | 15 | 0.667 | 1700 | 15 | 0.01 | 169.92 |
| Siteu | Tchouale 1 | 15 | 0.667 | 292 | 19 | 0.07 | 23.04 |
| Siteu | Le tchouala | 15 | 0.667 | 183 | 17 | 0.09 | 16.15 |
| Siteu | Tsinfem | 15 | 0.667 | 174 | 17 | 0.10 | 15.35 |
| Siteu | Haoussa | 15 | 0.667 | 143 | 15 | 0.10 | 14.30 |
| Siteu | Lefang | 15 | 0.667 | 86 | 12 | 0.14 | 10.75 |

**Table B. Malaria qPCR Detection Methods**

| ***Plasmodium falciparum (18s)*** | | | | |
| --- | --- | --- | --- | --- |
| **Adapted from:** | Veron V et al. Exp Parasitol 2009. 121(4):346-51. | | | |
| **Forward Primer** | 5' - ATTGCTTTTGAGAGGTTTTGTTACTTT - 3' | | | |
| **Reverse Primer** | 5' - GCTGTAGTATTCAAACACAATGAACTCAA - 3' | | | |
| **Probe** | 5' - FAM/ CATAACAGACGGGTAGTCAT /NFQ - 3' | | | |
| **Cycling conditions** | 1 cycle | 1 cycle | 45 cycles | |
| **Temperature (℃)** | 50 | 95 | 95 | 60 |
| **Time** | 2 min | 10 min | 15 s | 1 min |
| **Reaction conditions:** | PerfeCTa qPCR Tough Mix (QuantaBio, Beverly, MA, USA) | | | |
|  | Fwd primer | 300 nM | |  |
|  | Rev primer | 300 nM | |  |
|  | Probe | 200 nM | |  |
|  | Template DNA | 2 𝜇l | |  |
|  | Total volume | 12 𝜇l | |  |
| ***Plasmodium ovale spp.* (18s)** | | | | |
| **Adapted from:** | Mitchell C, et. al. Journal of Infectious Diseases. 2021 | | | |
| **Forward Primer** | 5’ - CCRACTAGGTTTTGGATGAAAVRTTTTT- 3’ | | | |
| **Reverse Primer** | 5’ - AACCCAAAGACTTTGATTTCTCATAA - 3’ | | | |
| **Probe** | 5’ - VIC/CRA AAGGAATTYTCTTATT /MGB- 3’ | | | |
| **Cycling conditions** | 1 cycle | 1 cycle | 45 cycles | |
| **Temp(℃)** | 50 | 95 | 95 | 52 |
| **Time** | 2 min | 10 min | 15 s | 1 min |
| **Reaction:** | PerfeCTa qPCR Tough Mix (QuantaBio, Beverly, MA, USA) | | | |
|  | Fwd primer | 400 nM | | |
|  | Rev primer | 400 nM | | |
|  | Probe | 200 nM | | |
|  | Template DNA | 2 𝜇l | | |
|  | Total volume | 12 𝜇l | | |
| ***Plasmodium vivax* (18s)** | | | | |
| **Adapted from:** | Brazeau N, et. al. Nature Communications. 2021 | | | |
| **Forward Primer** | 5’ - ACGCTTCTAGCTTAATCCACATAACT - 3’ | | | |
| **Reverse rimer** | 5’ - ATTTACTCAAAGTAACAAGGACTTCCAAGC - 3’ | | | |
| **Probe** | 5' - /56-FAM/TTCGTATCG/ZEN/ACTTTGTGCGCATTTTGC/3IABkFQ/ - 3' | | | |
| **Cycling conditions** | 1 cycle | 1 cycle | 45 cycles | |
| **Temperature (℃)** | 50 | 95 | 95 | 60 |
| **Time** | 2 min | 10 min | 15 s | 1 min |
| **Reaction:** | PerfeCTa qPCR Tough Mix (QuantaBio, Beverly, MA, USA) | | | |
|  | Fwd primer | 400 nM | | |
|  | Rev primer | 400 nM | | |
|  | Probe | 200 nM | | |
|  | Template DNA | 5 𝜇l | | |
|  | Total volume | 18 𝜇l | | |

**References:**

1. Veron V, Simon S, Carme B. Multiplex real-time PCR detection of P. falciparum, P. vivax and P. malariae in human blood samples. Exp Parasitol. 2009 Apr;121(4):346-51.

2. Gumbo A, Topazian HM, Mwanza A, Mitchell CL, Puerto-Meredith S, et al. Occurrence and Distribution of Nonfalciparum Malaria Parasite Species Among Adolescents and Adults in Malawi, Journal Infect Dis. 2022 Jan;225(2):257–268

3. Brazeau NF, Mitchell CL, Morgan AP et al*.* The epidemiology of *Plasmodium vivax* among adults in the Democratic Republic of the Congo. Nat Commun*.* 2021 Jul;12(1):4169.

**Table C. *P. falciparum* Gametocyte Detection by qPCR Methods**

| ***P. falciparum* male (*pfmget*)** | | | | |
| --- | --- | --- | --- | --- |
| **Forward Primer** | 5' – GGTCCAAATATAAAATCCTGTTC-3' | | | |
| **Reverse Primer** | 5' – TGTGTAACGTATGATTCATTTTC-3' | | | |
| **Probe** | 5' – FAM -5`CAGCTCCAGCATTAAAAACAC-BHQ1- 3' | | | |
| ***P. falciparum* female (*pfs25*)** | | | | |
| **Forward Primer** | GAA ATC CCG TTT CAT ACG CTT G | | | |
| **Reverse Primer** | AGT TTT AAC AGG ATT GCT TGT ATC TAA | | | |
| **Probe** | [AminoC6+HEX]-TGT AAG AAT GTA ACT TGT GGT AAC GGT-[BHQ1a~Q] | | | |
| **Duplexed *P. falciparum*** | | | | |
| **Cycling conditions** | 1 cycle | 1 cycle | 45 cycles | |
| **Temperature (℃)** | 50 | 95 | 95 | 59 |
| **Time** | 15 min | 2 min | 15 sec | 1 min |
| **Reagent** | **Volume (**𝜇**L)** | | | |
| **SuperScript III RT Platinum Taq Mix** | 0.5 | | | |
| **2X Reaction Mix with ROX** | 12.5 | | | |
| **pfs25 fwd primer (10** 𝜇**M)** | 2 | | | |
| **pfs25 rev primer (10** 𝜇**M)** | 2 | | | |
| **pfs25 probe (10** 𝜇**M)** | 0.2 | | | |
| **pfmget fwd primer (10** 𝜇**M)** | 2 | | | |
| **pfmget rev primer (10** 𝜇**M)** | 2 | | | |
| **pfmget probe (10** 𝜇**M)** | 0.2 | | | |
| **H2O** | 0.6 | | | |
| **Template RNA** | 3 | | | |
| **Total volume/reaction** | 25 | | | |

**Table D. *P. ovale spp* Gametocyte Detection by qPCR Methods**

| ***P. ovale* *mget ​​*(PocGH01_12044900_p1 & PowCR01_120040400_p1)** | | | | |
| --- | --- | --- | --- | --- |
| **Pf ortholog** | pfmGET | | | |
| **Forward Primer (P. ovale curtisi)** | CTTCGCATCCCCAGATTTCC | | | |
| **Reverse Primer (P. ovale curtisi)** | GCCATGTTTGCTTAATTGCCC | | | |
| **Probe** | FAM-AAAAGAAGC/ZEN/AAAGAACTCAAAGG-/3IABkFQ/ | | | |
| **Forward Primer (P. ovale walikeri)** | CGCATACCCAGAATTACCCC | | | |
| **Reverse Primer (P. ovale walikeri)** | GTCTCCTTCTCCTGCCTGAG | | | |
| **Cycling conditions** | 1 cycle | 1 cycle | 45 cycles | |
| **Temperature (℃)** | 50 | 95 | 95 | 55 |
| **Time** | 15 min | 2 min | 15 sec | 30 sec |
| **Reagent** | **Volume (uL)** | | | |
| **SS3 RT Platinum Taq Mix** | 0.5 | | | |
| **2X Reaction Mix with ROX** | 12.5 | | | |
| **poc_male forward (10** 𝜇**M)** | 2 | | | |
| **poc_male reverse (10** 𝜇**M)** | 2 | | | |
| **po_male probe (10** 𝜇**M)** | 0.4 | | | |
| **pow_male forward (10** 𝜇**M)** | 2 | | | |
| **pow_male reverse (10** 𝜇**M)** | 2 | | | |
| **H2O** | 0.6 | | | |
| **Template RNA/plasmid** | 3 | | | |
| **Total volume/reaction (**𝜇**L)** | 25 | | | |
| ***pos25*** | | | | |
| **Forward Primer** | CGTACCCGCTGAATGCAAAG | | | |
| **Reverse Primer** | GCCTATATTACATGAGCATCTACC | | | |
| **Probe** | FAM-AACCCAAGCCCGGATAAT–MGB –EQ | | | |
| **Cycling conditions** | 1 cycle | 1 cycle | 45 cycles | |
| **Temperature (℃)** | 55 | 95 | 95 | 58 |
| **Time** | 15 min | 1 in | 10 sec | 1 min |
| **Reagent** | **Volume (**𝜇**L)** | | | |
| **SS3 RT Platinum Taq Mix** | 0.5 | | | |
| **2X Reaction Mix with ROX** | 12.5 | | | |
| **Pos25 forward (10** 𝜇**M)** | 2 | | | |
| **Pos25 reverse (10** 𝜇**M)** | 2 | | | |
| **Pos25 probe (10** 𝜇**M)** | 0.6 | | | |
| **H2O** | 4.4 | | | |
| **Template RNA/plasmid** | 3 | | | |
| **Total volume/reaction (**𝜇**L)** | 25 | | | |

**Table E. Limit of detection for *P. ovale spp.* gametocyte assay**

| ***pomget*** | | |
| --- | --- | --- |
| **Copies/𝜇L** | **Replicates amplified** | **Replicates run** |
| 2871 | 12 | 12 |
| 236 | 11 | 12 |
| 47 | 2 | 12 |
| 9 | 0 | 12 |
| 2 | 0 | 12 |
| ***pos25*** | | |
| **Copies/𝜇L** | **Replicates amplified** | **Replicates run** |
| 200800 | 5 | 5 |
| 40160 | 5 | 5 |
| 8032 | 5 | 5 |
| 1606.4 | 5 | 5 |
| 321.3 | 5 | 5 |
| 64.3 | 5 | 5 |
| 12.9 | 4 | 5 |
| 2.6 | 3 | 5 |
| 0.5 | 0 | 5 |

**Table F. Interassay and intra-assay coefficient of variation (CV) for male *P. ovale* gametocyte detection using *pomget*.** Three serially diluted standards in triplicate across 5 different qPCR runs.

| **Intra-assay CV of *pomget*** | | | |
| --- | --- | --- | --- |
| **Transcript copies/**𝜇**L** | **Mean %CV of Ct** | **%CV IQR** | |
| 287100 | 1.45% | 0.96% - 2.05% | |
| 28710 | 2.07% | 0.69% - 2.81% | |
| 2871 | 1.36% | 0.50% - 2.27% | |
| **Interassay CV** | | | |
| **Transcript copies/**𝜇**L** | **Mean of means (Ct)** | **SD** | **%CV** |
| 287100 | 23.87 | 1.38 | 5.80% |
| 28710 | 29.82 | 1.45 | 4.87% |
| 2871 | 33.46 | 1.84 | 5.48% |

**Table G. Weighted bivariate and adjusted odds ratios with 95% CI**

| ***P. falciparum*** | **OR (95% CI)** | **p-value** | **aOR (95% CI)** | **p-value** |
| --- | --- | --- | --- | --- |
| Male v Female | 1.15 (0.85-1.57) | 0.36 | 1.04 (0.8-1.37) | 0.75 |
| Child v Adult | 1.53 (1.12-2.08) | 0.007 | 1.80 (1.25-2.58) | 0.002 |
| Used a bednet last night | 0.90 (0.67-1.23) | 0.51 | 0.96 (0.67-1.37) | 0.82 |
| Malaria Tx in last 28 days | 2.05 (1.38-3.04) | 0.0004 | 2.08 (1.27-3.39) | 0.004 |
| Daily exposure to river/creek | 1.57 (1.16-2.12) | 0.004 | 1.53 (0.97-2.44) | 0.07 |
| Household owns any livestock | 1.32 (0.95-1.83) | 0.098 | 1.58 (0.71-3.53) | 0.26 |
| Owns chickens | 1.01 (0.75-1.37) | 0.94 | 0.73 (0.32-1.64) | 0.43 |
| Owns cows | 0.16 (0.02-1.2) | 0.074 | 0.11 (0.01-1.41) | 0.09 |
| Owns goats | 1.05 (0.72-1.54) | 0.78 |  |  |
| Owns pigs | 1.02 (0.71-1.46) | 0.91 | 0.89 (0.51-1.56) | 0.67 |
| Glass window covering | 0.70 (0.5-0.97) | 0.034 | 0.76 (0.48-1.21) | 0.24 |
| Travel in CMR in last 28 days | 2.03 (1.44-2.86) | 0.0001 | 2.38 (1.66-3.4) | <.0001 |
| Travel outside CMR in last 28 days | 2.01 (0.25-15.98) | 0.51 | 2.44 (0.42-14.06) | 0.31 |
| ***P. ovale spp*** | **OR (95% CI)** | **p-value** | **aOR (95% CI)** | **p-value** |
| Male v Female | 0.92 (0.32-2.66) | 0.88 | 0.87 (0.48-1.58) | 0.65 |
| Child v Adult | 0.81 (0.27-2.42) | 0.70 | 1.03 (0.47-2.26) | 0.94 |
| Used a bednet last night | 0.94 (0.3-2.99) | 0.91 | 0.95 (0.31-2.9) | 0.93 |
| Malaria Tx in last 28 days | 2.00 (0.34-11.72) | 0.44 | 1.83 (0.26-12.74) | 0.53 |
| Daily exposure to river/creek | 1.05 (0.35-3.17) | 0.93 | 1.14 (0.43-3.05) | 0.78 |
| Household owns any livestock | 0.73 (0.22-2.43) | 0.60 | 0.21 (0.03-1.77) | 0.15 |
| Owns chickens | 1.07 (0.34-3.37) | 0.91 | 3.86 (0.77-19.24) | 0.098 |
| Owns goats | 0.05 (0.01-0.41) | 0.005 |  |  |
| Owns pigs | 1.17 (0.37-3.69) | 0.79 | 1.78 (0.89-3.55) | 0.099 |
| Glass window covering | 1.37 (0.48-3.85) | 0.56 | 1.19 (0.34-4.23) | 0.78 |
| Travel in CMR in last 28 days | 2.6 (0.87-7.75) | 0.086 | 2.53 (0.78-8.16) | 0.12 |

**Table H. *Anopheles* abundance in Dschang neighborhoods**

| **Health Area (number of households)** |  | **Median (IQR)** | **Mean (SD)** |
| --- | --- | --- | --- |
| **Bafou (n=4)** | *An. gambiae* | - | - |
|  | *An. funestus* | - | - |
|  | *An. nili* | - | - |
|  | *An. ziemanni* | 0.5 (0-1.5) | 1 (1.41) |
| **Fialah-Foréké (n=25)** | *An. gambiae* | 0 (0-1) | 0.6 (1.26) |
|  | *An. funestus* | - | - |
|  | *An. nili* | - | - |
|  | *An. ziemanni* | - | - |
| **Foto (n=35)** | *An. gambiae* | 1 (0-2) | 1.46 (2.59) |
|  | *An. funestus* | - | - |
|  | *An. nili* | - | - |
|  | *An. ziemanni* | 0 (0-0) | 0.086 (0.37) |
| **Siteu (n=37)** | *An. gambiae* | 1 (0-3) | 3.24 (5.54) |
|  | *An. funestus* | - | - |
|  | *An. nili* | - | - |
|  | *An. ziemanni* | **-** | **-** |

**Fig A. Distribution of parasitemias for *P. ovale spp*, *P. falciparum* and *P. vivax*.**

**
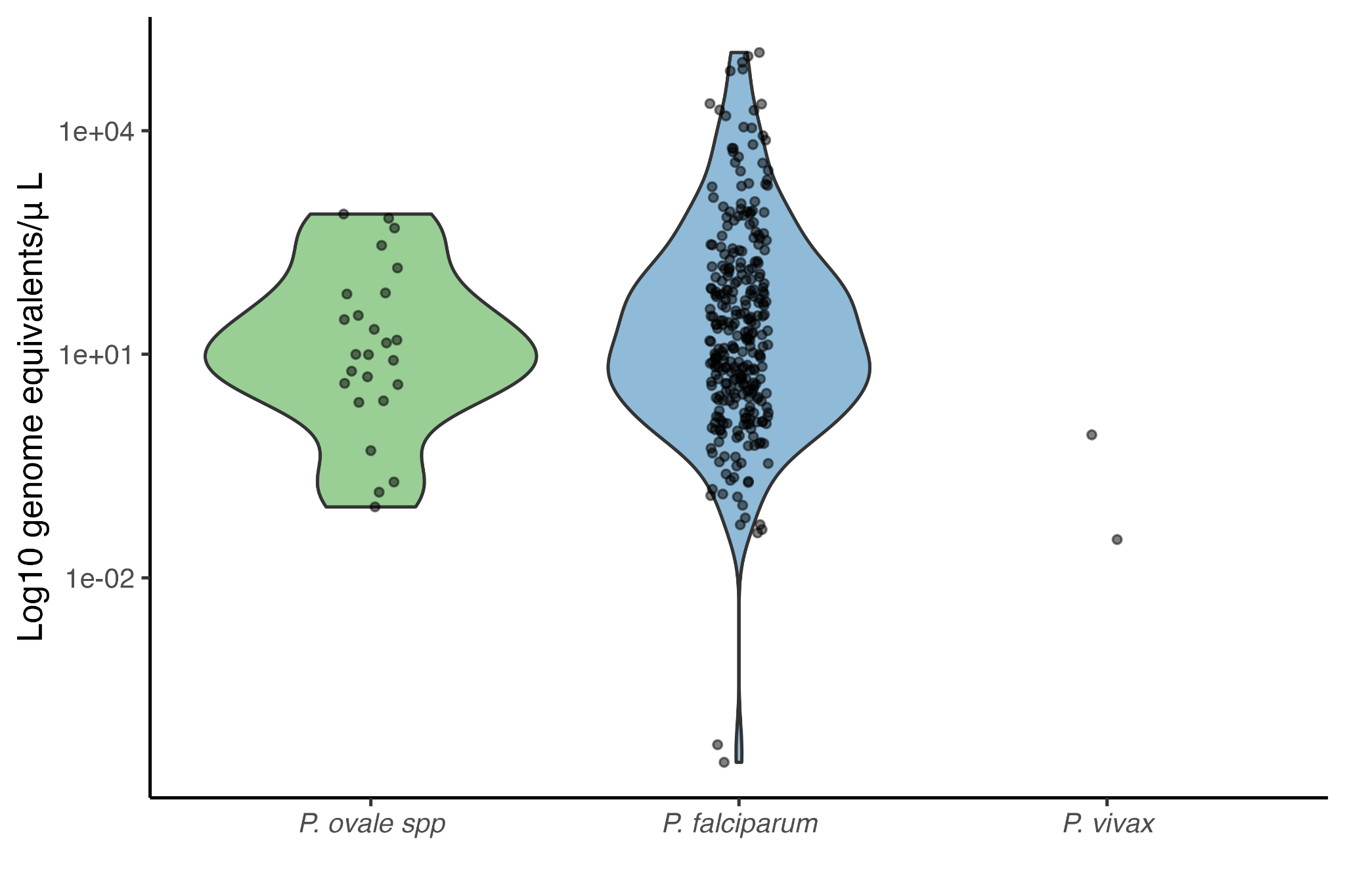
**

**Fig B. Correlation between gametocytemia and parasitemia in *P. falciparum*.**

**
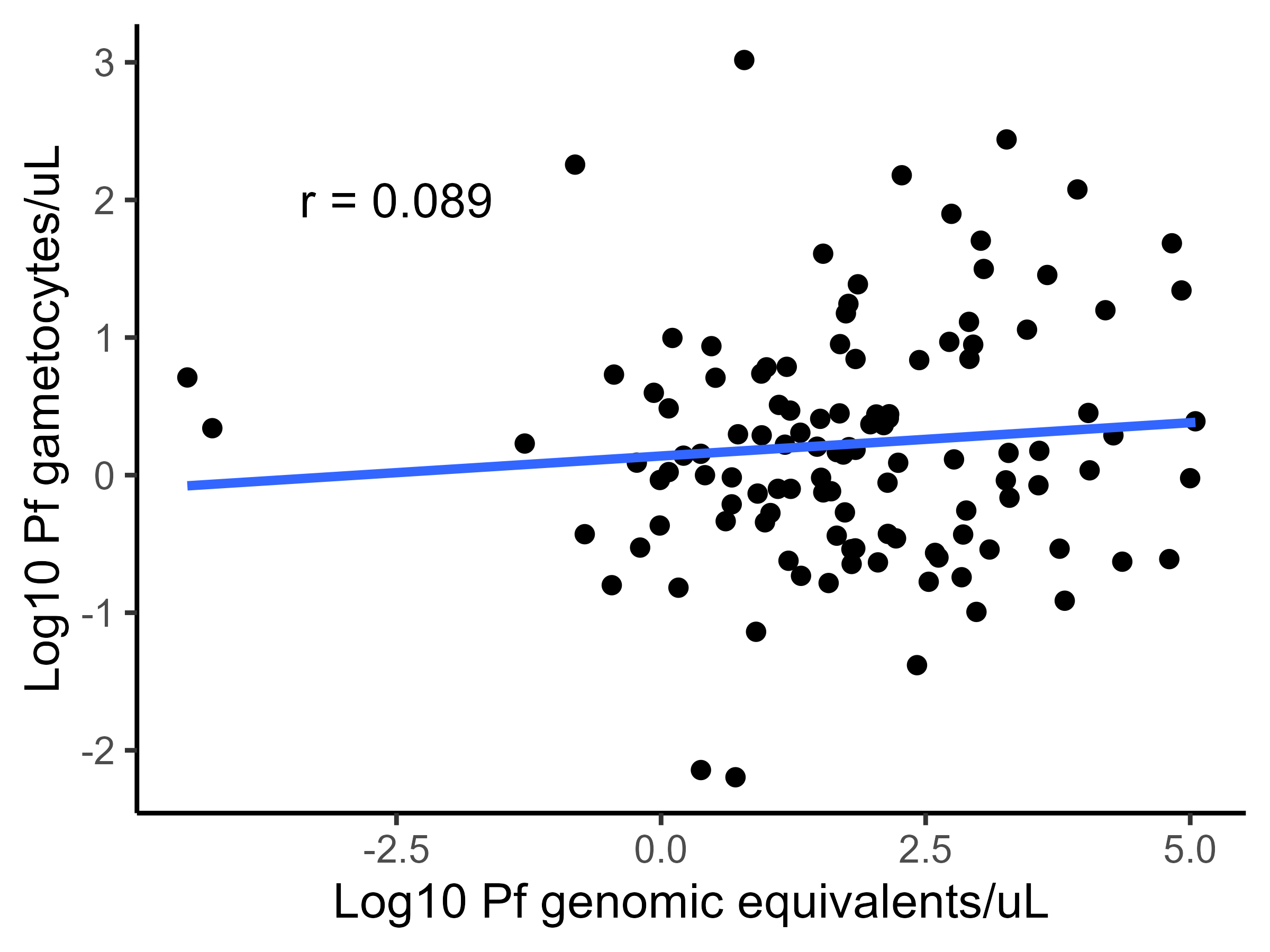
**

**Fig C. Limit of Detection (LOD) for *pomget* assay.**

A Probit analysis was conducted of replicate runs carried out using synthesized plasmids containing the *pomget* target sequence. An estimated LoD was calculated to be 281 transcript copies/uL (95% CI 162, 1310) based on the observed hit rate.

**
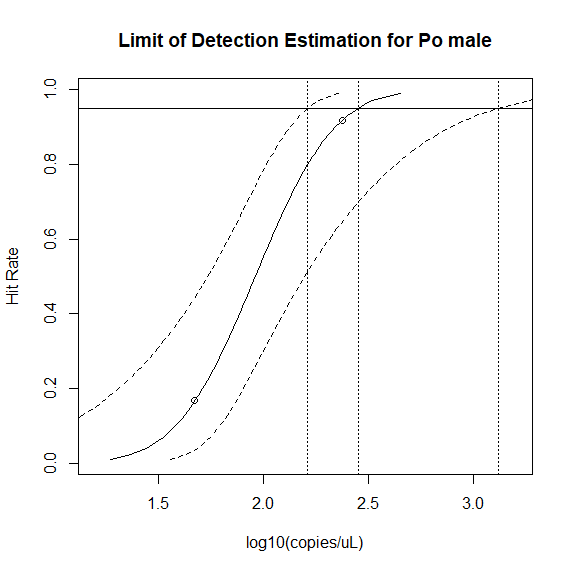
**

**Fig D. Mosquito capture time for indoor and outdoor collections across all households**

**
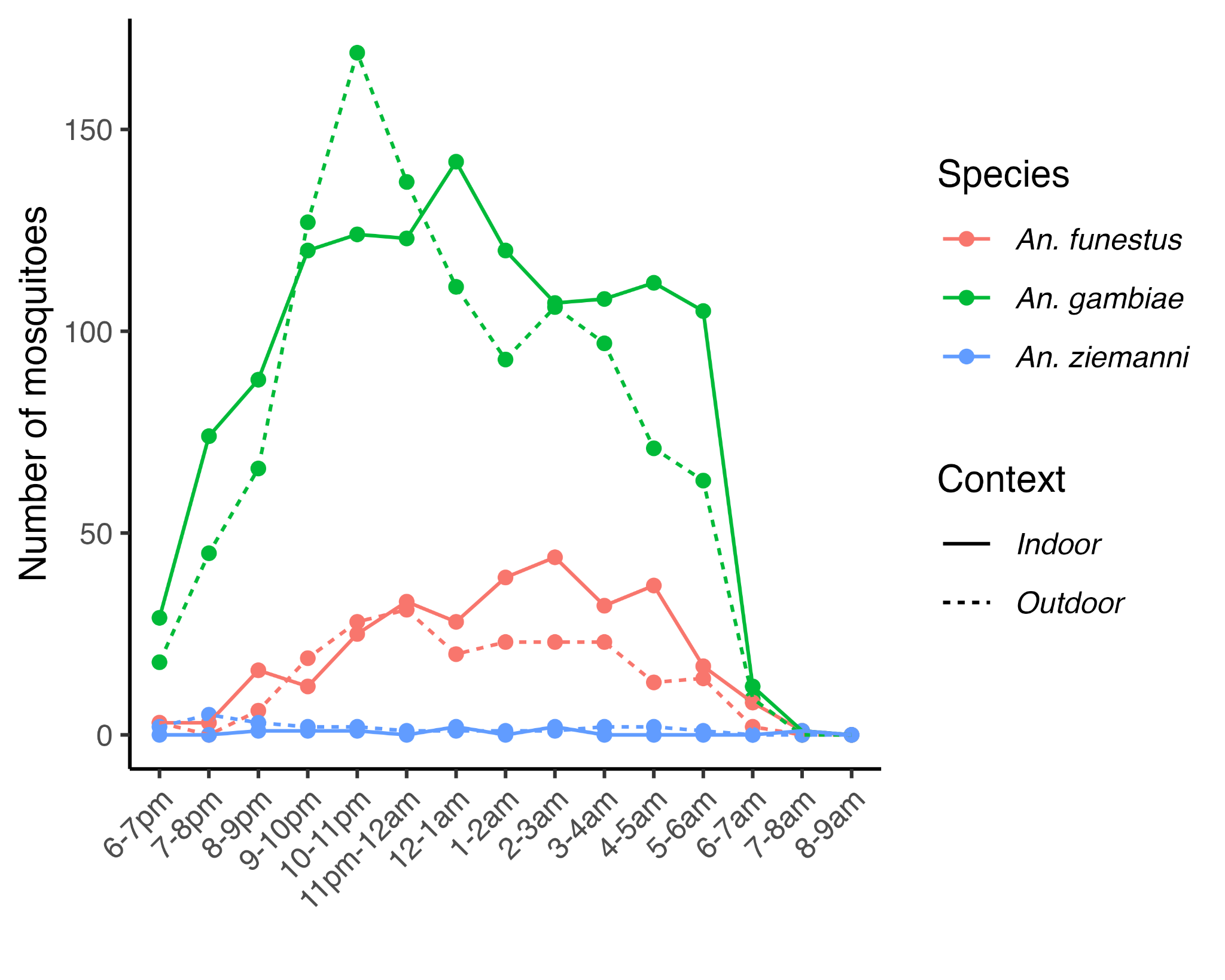
**
